# Clinical characteristics and risk factors predictive of pulmonary embolism complicated in bronchiectasis patients: a retrospective study

**DOI:** 10.1186/s12890-022-02016-9

**Published:** 2022-06-09

**Authors:** Tiantian Deng, Ke Xu, Beishou Wu, Fei Sheng, Xu Li, Zhuxian Zhu, Ziqiang Zhang

**Affiliations:** 1 Department of General Medicine, Tongji University School of Medicine, Shanghai, China; 2 Shanghai Nanxiang Community Health Service Center, Shanghai, China; 3 Department of Nephrology, Tongji Hospital, Tongji University School of Medicine, Shanghai, China; 4 Department of Infectious Disease, Tongji Hospital, Tongji University School of Medicine, Shanghai, China; 5 Department of Respiratory and Critical Care Medicine, Tongji Hospital, Tongji University School of Medicine, Shanghai, China

**Keywords:** Bronchiectasis, Pulmonary embolism, Hemoptysis, Clinical features, Risk factors

## Abstract

**Objective:**

Pulmonary embolism (PE) is a rare complication in bronchiectasis (BE) patients associated with a high rate of mortality and morbidity. However, data regarding bronchiectasis patients complicated with PE are limited. Early diagnosis of PE in bronchiectasis patients can improve the prognosis, this study aimed to investigate the clinical features and potential risk factors for early diagnosis of PE in bronchiectasis patients.

**Methods:**

Data of Patients were collected from Tongji Hospital of Tongji University of China. Bronchiectasis patients complicated with pulmonary embolism were named as BE/PE group (n = 63), as well as contemporaneous aged- and sex-matched bronchiectasis patients without pulmonary embolism named as BE group (n = 189), at a ratio of 1:3(cases to controls). Clinical parameters and risk factors were analyzed.

**Results:**

Univariate analysis shows that long-term bed rest, chronic lung disease, autoimmune disease, peripheral artery disease (PAD), tuberculosis history, dyspnea, blood homocysteine, CD4/CD8 ratio, or SIQIIITIII syndrome were closely correlated with the incidence of PE in the bronchiectasis patients (p < 0.05). Multivariate logistic regression analysis of significant variables showed that CD4/CD8 ratio (OR 1.409, 95% CI 1.045–1.901) and autoimmune disease (OR 0.264, 95% CI 0.133–0.524) are independent risk factors for BE/PE patients, compared with the BE patients. 53 out of 189 (28.0%) BE patients had hemoptysis, and 15 out of 63 (23.8%) BE/PE patients had hemoptysis (p > 0.05).

**Conclusions:**

The coexistence of pulmonary embolism and bronchiectasis are rarely encountered and easily to be ignored. Early identification of the clinical characteristic and potential risk factors of pulmonary embolism in bronchiectasis patients may help optimize the treatment strategies.

## Background

Bronchiectasis (BE) and pulmonary embolism (PE) may share similar risk factors, such as rheumatoid arthritis, inflammatory bowel disease, pneumonia, active malignancy tumor, or α1-antitrypsin [1–3]. Patients with bronchiectasis usually manifest as repeated cough, sputum, or hemoptysis. Unlike bronchiectasis, more common manifestations in patients with pulmonary embolism are chest tightness, chest pain, shortness of breath, or hemoptysis The symptoms of bronchiectasis and pulmonary embolism are sometimes overlapped with each other, such as hemoptysis, and usually not specific [2]. Therefore, the clinical manifestation of PE is easily ignored in patients with bronchiectasis.

Hemoptysis is a common symptom of bronchiectasis or pulmonary embolism could be fatal [4], however, the treatment strategies for these two diseases may be opposite. When hemoptysis due to bronchiectasis, hemostatic drugs are usually considered, while early anticoagulant therapy may be needed in pulmonary embolism patients even if hemoptysis occurs. Therefore, early identification of pulmonary embolism in bronchiectasis patients is extremely necessary.

So far, current clinical data concerning the comorbidity of bronchiectasis and pulmonary embolism is limited. To avoid missed diagnosis and help early identification of pulmonary embolism in bronchiectasis patients, this study tried to analyze the clinical features and potential risk factors of pulmonary embolism complicated in bronchiectasis patients.

## Methods

### Study design

Data of patients from Tongji Hospital of Tongji University of China from January 2009 to September 2021 were collected in this retrospective study. As a case–control study, sixty-three BE/PE patients (bronchiectasis patients complicated with PE) were identified, including 38 males and 25 females, and accounted for 1.2% of 4892 bronchiectasis patients and 5.5% of 1135 PE patients hospitalized over this study period. as well as contemporaneous aged- and sex-matched bronchiectasis patients without pulmonary embolism (BE group, n = 189) as control, at a ratio of 1:3(cases to controls), including 113 males and 76 females.

The diagnosis for bronchiectasis is mainly based on chest HRCT [5] and should be further clarified based on the previous medical history, clinical symptoms, signs, and laboratory results. CT pulmonary angiography (CTPA) or magnetic resonance pulmonary angiography is used to confirm the diagnosis of PE [2].

### Data collection

Clinical data were collected, including comorbidities such as peripheral arterial disease (PAD), chronic lung disease (COPD, asthma), history of tuberculosis, diabetes, coronary heart disease, chronic kidney disease, autoimmune disease; risk factors for venous thromboembolism (surgery, long term bed-rest, active malignancy tumor, trauma), Systolic blood pressure (SBP), Body mass index (BMI); clinical symptoms such as chest pain, cough, fever, dyspnea, hemoptysis; data of laboratory tests such as white blood cell count, neutrophil count, lymphocyte count, C-reactive protein, red blood cell volume, hemoglobin, platelet count, INR, PT, APTT, NT-pro-BNP, creating kinase, CD T cell series; electrocardiogram and deep venous thrombosis (DVT) detected by color Doppler ultrasound**.** PE-related in-hospital death was considered an adverse outcome. This study was approved by the Ethics Committee of Tongji Hospital of Tongji University (No. K-KYSB-2020-189). Informed consent was signed by the participants or their authorized family members.

### Statistical methods

Statistical analysis is performed by SPSS (NY, USA). Data are shown as medians with interquartile ranges (IQR) for continuous variables and numbers with percentages for categorical variables. The Mann–Whitney U test is used to compare continuous variables between the BE/PE and BE groups, and the chi-square test or Fisher’s exact test is used to compare the categorical variables. Significant independent variables in univariate analysis were included in the regression model for multivariate conditional logistic regression analysis. *p* < 0.05 is considered to be statistically significant.

## Results

In the total of 63 BE/PE patients, including 40 (63.5%) males and 23 (36.5%) females, PE was diagnosed in 28 (44.4%) patients concurrently with bronchiectasis, and 35 (55.5%) patients sequentially after the diagnosis of bronchiectasis. The median age of the BE/PE group is 63 (IQR 60–66) years old, and 10 (12.7%) patients are younger than 65 years old. Among the 63 BE/PE patients, 15 patients (23.8%) performed hemoptysis. 8 out of the 15 patients (53.3%) received anticoagulation therapy and stopped bleeding, 2 out of the 15 patients (11.1%) died of hemoptysis without timely anticoagulation. Five out of the 15 (33.3%) BE/PE patients with persistent hemoptysis were mainly due to bronchiectasis, which was confirmed by pulmonary angiography. All the BE patients were diagnosed with HRCT. All patients, including the control patients, enrolled in this study were checked with CTPA.

BE/PE and BE patients were matched by age, sex, and risks associated with these variables could not be estimated. There was no significant difference between the BE/PE and BE groups in surgery, trauma, tumor, SBP, diabetes, and chronic kidney disease. In contrast, cases in BE/PE group were more likely to be long-term bed rest (20.6% versus 3.2%, p < 0.05), PAD (73.0% versus 51.3%, p < 0.05), chronic lung disease (28.6% versus 16.0%, p < 0.05), coronary heart disease (41.3% versus 26.2%, p < 0.05), autoimmune disease (25.0% versus 9.6%, p < 0.05), history of tuberculosis (44.3% versus 24.6%, p < 0.05) and adverse outcome (6.3% versus 0%, p < 0.05). (Table 1).

**Table 1:** Baseline and clinical characteristics of the PE and BE/PE patients

Characteristics	BE/PE (n = 63)	BE (n = 189)	*P* value
Age			
≥ 65	53 (84.1)	159 (84.1)	Matched variable
< 65	10 (15.9)	12 (15.9)	Matched variable
Sex			
Male	40 (63.5)	120 (63.5)	Matched variable
Female	23 (36.5)	69 (36.5)	Matched variable
Ever-smoker	25 (39.7)	46 (24.3)	0.146
BMI			0.203
Underweight	7 (11.1)	21 (11.1)	
Normal	20 (31.7)	87 (46.9)	
Overweight	27 (42.9)	64 (33.9)	
Obesity	9 (14.3)	17 (9.0)	
Risk factor for VTE			
Surgery or trauma	5 (7.9)	9 (4.8)	0.348
Tumor	12 (19.0)	24 (12.8)	0.218
Long-term bed rest	13 (20.6)	6 (3.2)	0.024
*Comorbid condition*			
Chronic lung diseas disease^a^	18 (28.6)	30 (16.0)	0.029
Diabetes mellitus	14 (23.0)	27 (14.4)	0.120
Coronary heart disease	26 (41.3)	49 (26.2)	< 0.001
Chronic kidney disease	5 (9.3)	14 (7.6)	0.686
Autoimmune disease	15 (25.0)	18 (9.6)	0.002
Peripheral artery disease	46 (73.0)	97 (51.3)	0.003
History of Tuberculosis	27 (44.3)	46 (24.6)	0.003
Presenting manifestation			
Chest pain	9 (14.3)	29 (15.3)	0.839
Fever	8 (12.7)	36 (19.0)	0.250
Dyspnea	24 (38.1)	39 (20.6)	0.006
Hemoptysis	15 (23.8)	53 (28.0)	0.512
SIQIIITIII syndrome	9 (19.1)	4 (4.3)	0.010
T wave changes in chest leads	23 (45.8)	48 (25.4)	0.090
DVT	22 (46.8)	12 (12.8)	< 0.001
Heart rate > 110/min^b^	8 (12.7)	14 (7.4)	0.198
SBP < 90 mmHg	6 (9.5)	13 (6.9)	0.491
Adverse outcome	4 (6.3)	0 (0)	< 0.001

Data are presented as median (interquartile range) or n (%)

^a^Chronic lung disease includes chronic obstructive pulmonary disease, asthma

^b^Reference [6]

BMI, body mass index; VTE, venous thromboembolism; SBP, Systolic blood pressure. DVT, Deep venous thrombosis

SIQIIITIII syndrome was evaluated in the PE and BE/PE patients respectively, which was statistically significant (p < 0.05). While our results showed that there was no significant difference between the two groups in T wave changes in the chest leads 23(36.5%) vs. 48(25.4%) (p > 0.05), and heart rate (> 110/min) [6] 8(12.7%) vs. 14(7.4%) (p > 0.05) (Table 1).

The clinical manifestations of BE/PE and BE groups are shown in Table 1. Our results showed that there was no significant difference between the two groups in chest pain (14.3% versus15.3%, p > 0.05), fever (12.7% versus19.0%, p > 0.05), hemoptysis (23.8% versus28.0%, p > 0.05). While, there was a significant difference in the manifestation of dyspnea between the two groups (38.1% versus 20.6%, p < 0.05).

The levels of blood markers, including homocysteine (10.20 mmol/L vs. 11.00 mmol/L, p < 0.05) were significantly different between the BE/PE and the BE group. There was no significant difference in blood inflammation indicators between the BE/PE and BE groups, such as WBC count, neutrophil count, lymphocyte count, and CRP, which may be related to the progression of PE and formation of pulmonary embolism (Table 2).

**Table 2:** Clinical parameters of the PE and BE/PE patients

Item	BE/PE (n = 63)	n	BE (n = 189)	n	*P* value
WBC count (*109/L)	7.61 (6.50–9.00)	56	8.34 (7.07–9.66)	169	0.738
Neutrophil count (*109/L)	4.37 (3.3–5.5)	56	4.50 (3.24–5.59)	169	0.100
Lymphocyte count (*109/L)	1.64 (1.20–2.32)	56	1.40 (1.10–1.60)	169	0.400
CRP (mg/L)	12.98 (3.03–51.21)	54	45.57 (8.44–86.97)	166	0.481
Red blood cell volume (%)	37.1 (34.2–41.3)	56	37.10 (34.9–39.7)	169	0.100
Hemoglobin (g/L)	127 (120.0–134.0)	56	126 (118.00–133.5)	169	0.775
Platelet count (*109/L)	206 (186.0–254.0)	56	201 (153.00–218.00)	169	0.992
INR	0.98 (0.91–1.10)	50	0.97 (0.912–1.06)	158	0.583
PT(s)	11.10 (10.4–12.40)	50	11.10 (10.04–12.35)	158	0.895
APTT(s)	31.20 (28.70–34.80)	50	32.50 (28.60–46.08)	158	0.994
TnI (ng/ml)	0.17 (0.01–2.11)	58	0.024 (0.005–2.11)	164	0.085
proBNP (pg/ml)	81.8 (31.20–152.94)	49	83.40 (34.90–152.94)	167	0.710
Creatine Kinase (U/L)	59.0 (42.00–81.42)	49	59.5 (36.00–81.42)	156	0.247
Lactate dehydrogenase (U/L)	195.76 (168.0–211.0)	48	195.76 (160.00–199.50)	168	0.969
Triglycerides (mmol/L)	1.21 (0.88–1.30)	49	1.15 (0.76–1.21)	168	0.050
Total cholesterol (mml/L)	4.16 (3.48–4.85)	49	4.16 (3.82–4.60)	168	0.081
Low-density lipoprotein (mmol/L)	2.66 (2.29–2.92)	49	2.66 (2.33–3.25)	168	0.143
High-density lipoprotein (mmol/L)	1.23 (1.06–1.30)	49	1.12 (0.90–1.30)	168	0.105
Homocysteine (mmol/L)	14.20 (10.65–17.2)	61	12.60 (9.70–15.60)	164	< 0.001
Creatinine (μmol/L)	75.60 (64.00–84.00)	53	70.650 (52.50–92.25)	148	0.247
Total bile acid (μmol/L)	5.79 (3.10–8.40)	56	5.79 (2.50–5.79)	159	0.104
Procalcitonin (ng/ml)	4.299 (1.89–6.92)	50	3.79 (0.10–6.92)	160	0.362
NLR	2.62 (1.61–3.92)	56	2.95 (2.00–4.07)	169	0.364
Carbohydrate antigen 724 (U/mL)	2.73 (1.22–3.32)	47	2.47 (1.35–5.34)	146	0.352
CH50 (U/mL)	49.20 (32.60–52.42)	45	42.30 (39.90–43.40)	130	0.070

Data are presented as median (interquartile range) or n (%)

CRP, C-reactive protein; INR, International normalized ratio; PT, Plasma prothrombin time; APTT, Activated partial prothrombin time; NT-proBNP, N-terminal-pro-B-Type natriuretic peptide; NLR, Neutrophil to lymphocyte ratio

The median CD4/CD8 ratio in the BE/PE and PE group was 2.46 vs. 1.66, with a significant difference (P < 0.05). While, there were no significant differences in CD4, CD8, and CD19 T cell series between the two groups (p > 0.05) (Table 3).

**Table 3:** Laboratory examinations of T cell series between the PE and BE/PE patients

Item	BE/PE (n = 63)	n	BE (n = 189)	n	*P* value
CD3 + CD4 + (%)	31.00 (29.04–40.20)	53	30.44 (23.90–34.35)	120	0.721
CD19 + (%)	8.50 (6.20–21.10)	52	12.31 (8.39–15.25)	138	0.986
CD3 + CD8 + (%)	21.87 (17.20–22.00)	58	21.50 (16.40–27.60)	143	0.468
CD4/CD8	2.46 (1.62–2.56)	56	1.66 (1.10–2.68)	154	0.022

Data are presented as median (interquartile range) or n (%)

The univariate analysis showed that long-term bed-rest, chronic lung disease, coronary heart disease, autoimmune disease, peripheral artery disease, history of tuberculosis, along with homocysteine and CD4/CD8 ratio were statistically significant, as shown in Tables 1, 2 and 3. These variables were selected as the covariates and the multi-factor conditional logistic regression analysis was performed by using the stepwise forward method. Our results show that CD4/CD8 ratio (OR 1.409, 95% CI 1.045–1.901, p = 0.025) and autoimmune disease (OR 0.264, 95% CI 0.133–0.524, p < 0.001) are independent risk factors for the bronchiectasis patients complicated with pulmonary embolism, but not homocysteine (OR 0.999, 95% CI 0.994–1.005, p = 0.827) (Table 4).

**Table 4:** Multivariate conditional logistic regression analysis the PE and BE/PE patients

Influencing factors	OR	95% CI	*P* value
CD4/CD8	1.409	1.405–1.901	0.025
Autoimmune disease	0.264	0.133–0.524	< 0.001
Long-term bed rest	0.531	0.269–1.051	0.069
Peripheral artery disease	0.970	0.538–1.748	0.918
Coronary heart disease	0.713	0.414–1.228	0.223
History of Tuberculosis	1.288	0.694–2.389	0.422
Homocysteine	0.999	0.994–1.005	0.827
Chronic lung disease	0.566	0.315–1.016	0.056

*OR* odds ratio, *CI* confidence interval

## Discussion

The risk factor of pulmonary embolism refers to any factor that can lead to venous blood stasis, venous system endothelial damage, and blood hypercoagulability. Bronchiectasis patients coexisting with pulmonary embolism may be related to their common characteristics: (i) The onset or exacerbation of bronchiectasis is due to inflammation of the airway or systemic inflammation. Respiratory tract infection itself can increase the risk of pulmonary embolism [7–9]. (ii) Neutrophils dominated inflammation can generate ROS to eliminate bacteria [10, 11]. While excessive ROS release can cause damage to vascular endothelial cells, tissue factor exposure to the vascular wall, and promote thrombosis [12, 13]. (iii) Repeated acute exacerbations of bronchiectasis affect lung function and being in a state of chronic hypoxia, resulting in increased blood viscosity. The above characteristics as risk factors, which are more likely to lead to PE in bronchiectasis patients.

The clinical symptoms of PE are diverse and non-specific, ranging from asymptomatic to hemodynamic instability and sudden death. A study has found that 73% of patients with acute PE have dyspnea, 30% of patients have tachycardia, and 66% of patients have chest pain [14]. Regarding the clinical manifestations of bronchiectasis overlapped with pulmonary embolism, it is difficult to distinguish whether it is the exacerbation of bronchiectasis or a combination of pulmonary embolism. Our result showed it was different in the incidence of dyspnea (p < 0.05), 24(38.1%) cases in the BE/PE group and 39(20.6%) in the BE group had dyspnea. There was no significant difference in the incidence of chest pain, fever, and hemoptysis (p > 0.05). So, when bronchiectasis patients who show dyspnea cannot be explained by the primary disease, the possibility of PE should be early considered.

Long-term bed-rest is an important risk factor for pulmonary embolism [15, 16]. In our study of the univariate analysis, 13(20.6%) patients in the BE/PE group had a history of long-term bed-rest, while 6 (3.2%) patients in the BE group, with a significant difference.

Coronary atherosclerotic heart disease and peripheral artery disease both are manifestations of persistent vascular inflammation [17]. In this study, the univariate analysis showed coronary heart disease and peripheral artery disease are likely to be risk factors for bronchiectasis patients complicated with pulmonary embolism. Moreover, we compared the homocysteine levels between the patients with or without peripheral or coronary vascular disease. Our results showed that the median homocysteine levels in patients with or without peripheral artery disease were 13.6, and 12.55 (mmol/L) (p = 0.43). In patients with or without coronary vascular disease, it was 12.94 and 13.0 (mmol/L) (p = 0.97). Thus, although the prevalence of peripheral and coronary vascular diseases is very high in both PE patients and controls, it is likely not the main reason for the elevated levels of homocysteine. Furthermore, pulmonary embolism is one of the most common complications of active malignancy tumors [18]. However, our results of the univariate analysis show that the active malignancy tumor is not an independent risk factor for pulmonary embolism in bronchiectasis patients (p = 0.218).

Misdiagnosis and missed diagnosis and delayed anticoagulation in PE patients will threaten the patient's life. In our study, only 8 out of 15 hemoptysis patients (55%) received timely anticoagulation treatment, and 2 out of 15 patients (11%) died of hemoptysis without immediate anticoagulation. four patients had adverse outcomes due to the missed diagnosis of pulmonary embolism. Anticoagulant therapy is one of the integral parts of treatment in cases of pulmonary embolism (PE) that develops in other diseases [4]. Moreover, the IVC filter is a potential temporizing measure for the bleeding caused by pulmonary embolism. However, the issue of how to act when PE is reported in bronchiectasis patients manifested as hemoptysis may pose a real therapeutic challenge because these two conditions require a pharmacologically opposite nature of treatment [19]. Anticoagulant therapy was required to prevent further thrombosis while ongoing hemoptysis required procoagulant therapy. We should concern about the secondary bleeding caused by anticoagulation treatment, however, so far there is no specific guideline on the safety regarding anticoagulation in patients manifesting with pulmonary embolus and significant hemoptysis [20]. Anticoagulation is necessary for patients with pulmonary embolism, it prevents early mortality death [21]. However, anticoagulation is prohibited in patients with severe hemoptysis as it may cause fatal hemorrhage. Therefore, it needs further exploration for the management of such challenges.

The classic SIQIIITIII syndrome only occurs in 15–25% of patients with pulmonary embolism [22]. We compared the incidence of this syndrome by the electrocardiogram and found 9 (19.1%) cases in the BE/PE group and 4(4.3%) cases in the BE group performed SIQIIITIII syndrome, with a significant difference (p < 0.05). We also analyzed homocysteine in univariate analysis, and our result showed the homocysteine level had a significant difference in univariate analysis between the BE/PE group and the BE group (p < 0.05). However, further multivariate logistic regression analysis showed that the increased homocysteine level was not an independent risk factor for the prediction of BE/PE (p = 0.353).

Studies have shown that 87–97% of patients with pulmonary embolism have emboli from DVT [23]. For COPD patients, the GOLD guidelines recommend that all hospitalized patients with acute exacerbations of COPD should undergo routine lower extremity venous Doppler ultrasonography to determine if there is a venous thrombosis in the lower limbs [24]. However, there is still no recommendation for Doppler ultrasonography examination for patients with exacerbation of bronchiectasis. Our results showed that 22(46.8%) patients in the BE/PE group and 12(12.8%) patients in the BE group had DVT detected by color Doppler ultrasound, with a significant difference (p < 0.05). So, for patients with exacerbation of bronchiectasis, especially those who are complicated with dyspnea, lower extremity venous Doppler ultrasonography is necessary for assisting the diagnosis of PE.

Studies have revealed that autoimmune diseases are related to an increased risk of pulmonary embolism [25, 26]. Our results showed that the proportion of patients with the autoimmune disease in the BE/PE group was 25.0%, which was significantly higher than that in the BE group (9.6%) (p = 0.002). Further multivariate analysis showed that autoimmune disease (OR 0.264, 95% CI 0.133–0.524, p < 0.001) is likely to be an independent risk factor for bronchiectasis patients complicated with pulmonary embolism. Therefore, for bronchiectasis patients with autoimmune diseases, it is necessary to pay attention to the screen for pulmonary embolism. However, only a quarter of the patients with PE had an autoimmune disease in this study, so it is still hard to conclude the whole group. Further prospective studies are essential to identify the predictive value of inflammatory markers in this type of patient for predicting pulmonary embolism.

Studies have shown that immune and inflammatory factors are involved in the development of venous and arterial thrombosis [8, 27]. There are compromised T-cell immunity in patients with pulmonary hypertension and T-cell mediated immune deficiency in patients with chronic thrombotic pulmonary hypertension (CTEPH) [28, 29]. Furthermore, CD4/CD8 T ratio has been used as a clinical parameter to evaluate the patient’s immune function, CD4/CD8 ratio might be a predictor of immune activation and immune senescence [30]. However, various diseases can influence this parameter. Studies have suggested that infections due to different kinds of pathogenic microorganisms may cause different clinical types and outcomes of VTE. A study has shown that CD8 T cells significantly decreased while CD4/CD8 ratio significantly increased in VTE patients complicated with infection [31]. While CD4/CD8 may be affected by a variety of factors. CD4/CD8 ratio of less than 1.0 should be aggressively investigated for HIV infection as well as other causes of immune deficiency [32]. A Meta-analysis showed a significant decrease of CD4/CD8 ratio in brucellosis-infected patients, compared to healthy subjects [33]. Systematic and meta-analysis suggested there was a decreased CD4/CD8 ratio in the peripheral blood of pulmonary tuberculosis patients [34]. Moreover, CD4/CD8 ratio may be related to coronary plaque instability in unstable angina pectoris patients [35]. Our results showed the median CD4/CD8 ratio in the BE/PE patients and the BE patients was 2.46 and 1.66 respectively, with a significant difference (p = 0.022). Further multivariate analysis showed that CD4/CD8 ratio (OR 1.409, 95% CI 1.045–1.901, p = 0.0254) is likely to be an independent risk factor for bronchiectasis patients complicated with pulmonary embolism. We speculate that the increased CD4/CD8 ratio may be related to the activated immune and inflammatory state in BE/PE patients and the occurrence of pulmonary embolism may change the original CD4/CD8 ratio in patients with bronchiectasis. However, more prospective clinical studies are still needed. For example, we will explore whether the CD4/CD8 ratio in BE/PE rises and fall abruptly with the PE in future studies.

Our results showed that chronic lung disease is an independent risk factor for pulmonary embolism in patients with bronchiectasis (p = 0.029), so lower FEV1, diffuse bronchiectasis along with declined lung function may contribute to an elevated risk of pulmonary embolism in bronchiectasis patients. Rheumatoid arthritis complicated with bronchiectasis is usually characterized by decreased lung function, and increased mortality rate, and may be related to the development of pulmonary embolism. Moreover, hormones and disease-modifying anti-rheumatic drugs (DMARDs) are usually required to control this disease [36]. While hormones and DMARDs likely increase the risk of thromboembolism [36]. Patients with bronchiectasis are often accompanied by the colonization of Pseudomonas aeruginosa in the bronchus. While the drug resistance of Pseudomonas aeruginosa is closely related to the repeated exacerbation of bronchiectasis infection, which increases the risk of pulmonary embolism [37].

## Conclusions

The coexistence of bronchiectasis and pulmonary embolism is easy to be ignored in clinical practice, especially without the presence of factors that could provoke venous thromboembolism. Autoimmune disease or increased CD4/CD8 ratio likely has a predictive value for the diagnosis of bronchiectasis patients complicated with pulmonary embolism. Early identification of the characteristic clinical features and biomarkers of pulmonary embolism complicated in bronchiectasis patients is crucial for the early PE diagnosis and optimizing the treatment strategies.

## Data Availability

The datasets used and analyzed during the current study available from the corresponding author on reasonable request.

## Abbreviations

PE: Pulmonary embolism
BE: Bronchiectasis
BMI: Body mass index
PAD: Peripheral arterial disease
VTE: Venous Thromboembolism
SBP: Systolic blood pressure
CRP: C-reactive protein
INR: International normalized ratio
PT: Plasma prothrombin time
APTT: Activated partial prothrombin time
NT-proBNP: N-terminal-pro-B-Type natriuretic peptide
DVT: Deep venous thrombosis
